# Modulation of human airway smooth muscle biology by human adipocytes

**DOI:** 10.1186/s12931-018-0741-z

**Published:** 2018-02-27

**Authors:** Amanda Giesler, Manali Mukherjee, Katherine Radford, Luke Janssen, Parameswaran Nair

**Affiliations:** Firestone Institute for Respiratory Health, St Joseph’s Healthcare & Department of Medicine, McMaster University, 50 Charlton Avenue East, Hamilton, ON L8N 4A6 Canada

**Keywords:** Asthma, Obesity, Airway hyperresonponsiveness, Adipocytes, Airway smooth muscle

## Abstract

**Background:**

Asthma and obesity, two growing epidemics worldwide, may share an underlying causal relationship. Airway hyperresponsiveness (AHR), a defining component of asthma, has been documented in both ‘obese’ animal models and non-asthmatic obese individuals. However, there is a paucity of evidence that obesity-derived factors directly affect human airway smooth muscles (ASM).

**Methods:**

Experiments were designed with primary ASM and adipocytes isolated from the same human tissue explants (*n* = 6). The modulatory effects of human adipocytes extracted from subcutaneous (extrathoracic) and visceral (intrathoracic) depots, on ASM biology was examined with respect to proliferation, migration, contractility and pro-inflammatory cytokine synthesis.

**Results:**

Adipocyte-conditioned media as well as myocyte-adipocyte co-cultures failed to show any significant changes in the proliferative or migrational properties of the ASM. Adipocyte-conditioned media also had no effect on the contractility or relaxation of bovine tracheal muscle strips. In contrast, there was a moderate yet significant increase of IL-6 and eotaxin release by ASM incubated with adipocyte-conditioned media (*P* = 0.0035 and *P* = 0.0067, vs. control, respectively), thereby further consolidating the altered inflammatory state reported for both diseases.

**Conclusion:**

We report, for the first time, that adipocytes from either subcutaneous or visceral depots can trigger an inflammatory state in the ASM, with negligible modulatory effects on hyperplasia, hypertrophy or contractile properties.

**Electronic supplementary material:**

The online version of this article (10.1186/s12931-018-0741-z) contains supplementary material, which is available to authorized users.

## Background

An increase in incidence trends for two major chronic diseases, asthma and obesity, and a plausible ‘association’ thereof was premised as early as 1999 [1]. Epidemiological and fundamental research since then has confirmed asthma to be a major risk factor in obese individuals [2–5], and yet an ambiguity remains in defining a definite ‘causal’ relationship. Rasmussen and Hancox [6] in a recent review assessed the available literature on asthma and obesity, summarizing three plausible mechanisms that could explain the observed associations between the two chronic conditions; viz., (i) altered breathing mechanics in obese that contribute to the airway hyperresponsiveness (AHR), (ii) an underlying inflammatory pathway driven by adipose-secreted mediators or ‘adipokines’, and finally (iii) lifestyle changes contributing to gene-environmental interactions in those already pre-disposed to asthma. Several experimental animal (mouse) models of obesity exhibit adipokines to contribute to the airway inflammation antecedent to development of an asthma-like phenotype or trigger a heightened response post stimuli (reviewed in [7, 8]. On the contrary, data in humans, investigating the same association is less conclusive, [4, 6, 8, 9] and at times, conflicting [10–12].

Though airway inflammation or ‘bronchitis’ is considered responsible for the physiologic consequences of asthma, it is the AHR that distinguishes it from other obstructive lung disorders [13, 14]. Increased AHR has been reported in non-asthmatic obese individuals [15] and in teenagers with high body mass index (BMI) [16]. Indeed, while discussing their experimental diet-induced mouse models [17], Shore et al., commented that innate AHR was a characteristic feature of obese mice independent of the modality of obesity induction. A recent study by Al-Alwan and co-workers [18], reported that the inverse relation between adipose mass with lung function in obese asthmatics is attributed to the diminished respiratory compliance (compressed lungs in obese). But as rightly pointed out by Robert H. Brown [19] the study failed to address the real question: why obese individuals are at risk to develop asthma?

In the past 15 years, the focal point of asthma-obesity research has been centred on investigating the inflammatory profile common to both diseases as the ‘causal’ link. The release of adipokines from adipose stores along with increase in pro-inflammatory mediators, a phenomenon termed ‘metabolic inflammation’, till date remains responsible for the obesity-derived systemic complications like type 2 diabetes, staetohepatitis and metabolic syndrome (30). Consequent studies have confirmed that obese individuals have increased levels of adipokines and increased markers of systemic inflammation (like C-reactive protein) which positively correlates to asthma development and severity (6, 9). IL-6 and TNF-α govern the chronic low-lying systemic inflammation characteristic of obesity by paracrine inhibition of the anti-inflammatory adipokine “adiponectin” (8, 9, 33). Indeed, sputum adiponectin levels are lower in asthmatics compared to healthy, and high sputum adiponectin was computed with lower odds of asthma development, implying an altered inflammatory state in the lungs to be the causal link (34). On the contrary, Sidleva et al., reported a 12-month observation study of obese asthmatic women post-bariatric surgery, where the extent of reduction in adiposity was directly associated with reduced AHR, independent of the airway inflammation (12).

It is evident from animal models, that obesity can induce AHR via immunological mechanisms [7, 8]. Shore et al., reported that leptin, a pro-inflammatory adipokine directly correlated with the increased risk of asthma in obese [6, 9, 20], when exogenously administered to ovalbumin-sensitized mice augments induction of AHR [21]. This is in contrast to their subsequent studies where leptin-deficient obese mouse models developed innate AHR [7, 22]. Again, diet-induced obese mice developed innate AHR, which was dependent on the amount of time the obesity existed rather than its onset or the body mass [17]. The study showed a distinct effect of time on the inflammatory profile, where a significant quantity of serum leptin was measured in 30-week-old animals (compared to 22-week-old animals), which coincided with the measured AHR. Since human ASM cells express the receptor for leptin [23], a recent study by our research group showed exogenous addition of leptin to ASM, in vitro, inhibits smooth muscle migration, proliferation and IL-13-induced eotaxin. Thus, in contrast to the mouse models, it is unlikely that leptin-induced inflammation be the causal factor in the asthma-obesity relationship. In accordance, a population-based birth cohort study from New Zealand failed to show any statistical significance between serum leptin and clinical markers of asthma [10].

Besides leptin and adiponectin, adipokines consist of an array of mediators produced by adipocytes that include vinfastin, resistin, adipsin, in addition to eicasanoids and cytokines like IL-6, eotaxin and tumour necrosis factor alpha (TNFα), all of which have the potential to affect the ASM biology, dependent/independent of leptin [9]. There is no evidence to indicate that increased secretion of adipokines from adipose mass from human subjects could modulate ASM biology into a diseased phenotype distinctive of asthma, characterised by hyperplasia, hypertrophy and hyperreactivity [24]. Therefore, our current study was designed to identify a disease-defining role of adipocytes on ASM by engaging the entire array of mediators released, instead of segregating the effect of only “leptin” or “adiponectin”. Additionally, since visceral adipose tissue has been previously documented to be the main source of adiponectin [25], along with a variation in leptin gene expression between the adipose sites [26]; the study design also attempted to differentiate the effect of adipocytes isolated from different anatomical fat depots i.e. subcutaneous (extrathoracic) and visceral (intrathoracic), on ASM biology. The current study thus aimed to investigate the direct effect of adipocytes on ASM in an in vitro system, without the influence of circulating immune cells and other in vivo factors.

## Methods

All human samples used in the study had informed consent from respective patients and approval (#08–3085) from the Hamilton Integrated Research Ethics Board, St. Joseph’s Healthcare (HiREB, Hamilton, Ontario, Canada).

### Human adipocyte culture

Intrathoracic adipose tissue samples harvested from the mediastinum while extra-thoracic samples from the chest wall of the same patient, were collected during scheduled thoracic surgeries (refer to Table 1 for patient characteristics), and were processed separately (in parallel), as described [27]. Isolated adipocytes were cultured with 1% FBS-supplemented media, until 80% confluence was attained. Thereafter, they were maintained in serum-free adipocyte media for 3 days. The conditioned supernatants were used for ASMC stimulation or stored at -80 °C for future experiments. Regular quality control checks were performed for assessing purity of the cells acquired through these established in vitro protocols.

**Table 1:** Subject Characteristics

Total number of patients who donated tissue, n	7
Migration experiments, n	6
Other experiments, n	6
Males/Females	2/4
Height, cm (mean ± SD)	166 ± 7.5
Body mass index (mean ± SD)	32.9 ± 6.1
Age in years (mean ± SD)	69.5 ± 13.3
FEV_1_, % predicted	75.33 ± 22

*N.B.* FEV_1_ – forced expiratory volume in 1 s; SD- standard deviation; all donors were of Caucasian ethnicity. Tissue from one donor lost to contamination, demographic data reported for 6

### Human adipocyte and ASM co-culture

Smooth muscle tissue isolated from macroscopically disease-free areas of human bronchi (obtained from lung tissues resected for cancer), were grown to confluence as previously described [28]. Experiments were conducted between passages 2–4. Human ASM cells (ASMC) were seeded at 10^5^ cells/mL in the basolateral chamber of the Transwell^®^ inserts (3.0 μm pore-size, Corning Life Sciences, Tewksbury, MA) and maintained in 10% FBS-supplemented media for 3 days [28]. Thereafter, isolated adipocytes from both sites [27], were seeded separately on the apical chambers; the co-culture hereon was maintained in 1% FBS supplemented adipocyte media.

### Proliferation assays

The proliferation of ASMCs was assessed by cell-counting as described elsewhere [29]. Briefly, ASMCs were seeded at 10^5^ cells/mL in a 24 well-plate maintained in 10% FBS RPMI for 24 h, following which a synchronization of the cell-cycle was allowed in serum-free conditions for 24 h. Subsequently, the ASMCs were challenged with either control or adipocyte-conditioned media for four days before counting using trypan blue exclusion method.

### Migration assays

The effect of adipocyte-released mediators on the migrational property of ASMCs was assessed by Transwell® based migration protocol as previously described [28]. Briefly, ASMCs serum-starved for 24 h, were seeded on to the apical chambers of a collagen-coated 8.0 μm pore Transwell® plate containing adipocyte-conditioned media in the basolateral chamber. The number of migrated cells stuck on the lower surface of the insert was counted and processed. 10 ng/mL PDGF was used as a positive control as per previous reports [23, 28].

### Bovine tracheal muscle bath techniques

Isometric contractility studies were performed with bovine tracheal strips standardized with 60 mM KCl [23]. It was made sure that the tracheal strips did not have any residual traces of epithelium attached. The tissues were primed with adipocyte-conditioned media (or control) diluted (1:2) in Krebs-Ringer’s solution for 20 min and treated subsequently with either increasing strengths of carbachol (10^− 10^ – 10^− 5^ M), or isoproterenol (10^− 10^ – 10^− 5^ M). Each dose was added when the previous dose reached a plateau. After the last dose, the tissues were washed and a final KCl challenge was performed to verify the continued viability of the tissues.

### Cytokine analysis

A Bio-Plex Pro Bead Assay (Bio-Rad, Mississauga, ON) was used to measure IL-6, TNF-α and eotaxin with results analysed on Bio-Plex Manager 6.0 Software (Bio-Rad Laboratories Inc., Mississauga, ON). The limits of detection were 1.54, 1.51 and 4.75 pg/mL for IL-6, eotaxin and TNF-α respectively.

### Statistics

Comparisons of proliferation, migration and cytokine synthesis between the tested conditions were assessed using repeated measures analyses of variance (ANOVA) on GraphPad Prism version 7.0 (La Jolla, CA, USA), unless mentioned otherwise. Dose-response curves were constructed using non-linear regression. Results were expressed as mean ± standard error (SEM) from *n* = 2 technical repeats from samples collected from 6 individual donors. A *P* value < 0.05 was considered as significant.

## Results

### Airway smooth muscle cell proliferation

**Fig. 1:**
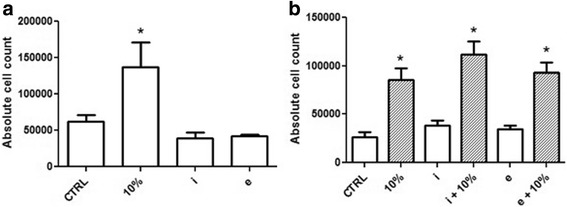
Effect of adipokines on the proliferative capacity of ASMCs. **a** ASMC counts expressed as number of cells in presence of serum-free adipocyte media (CTRL), 10% FBS supplemented media (10%), intrathoracic (*i*) and extrathoracic (*e*) adipocyte conditioned media. Data represented as mean cell count ± SEM; *P = 0.0008, *n* = 6 (donors); **b** ASMCs co-cultured for 3 days with intrathoracic (*i*) and extrathoracic (*e*) human adipocytes ±10% FBS supplemented RPMI. ASMCs grown in cell-free, non-conditioned serum-free adipocyte media used as control (CTRL). Data represented as mean cell count ± SEM. RM-ANOVA, Bonferroni comparison * *P* < 0.0001, compared to control, i & e, *n* = 6 (donors)

The proliferation of human ASMCs was significantly increased in the presence of 10% FBS (*P* = 0.0008 vs. serum-free control), while the intra-thoracic and extra-thoracic ‘conditioned’ media had no effect on their growth (Fig. 1a). Further on, though co-culture with intrathoracic and extrathoracic adipocytes in 10% FBS supplemented media showed significant increase in ASMC cell count from serum-free conditions (*P* < 0.0001, Fig. 1b), it failed to show any significant increase in the proliferation rate when grown in absence of adipocytes (10% FBS media, Fig. 1b). In addition, there was no statistical significance between the two depots, extra-thoracic and intra-thoracic, in either experimental set-up (Fig. 1), indicating that adipocytes or their secreted mediators have limited effect on ASMC proliferation.

### Airway smooth muscle cell chemotaxis

**Fig. 2:**
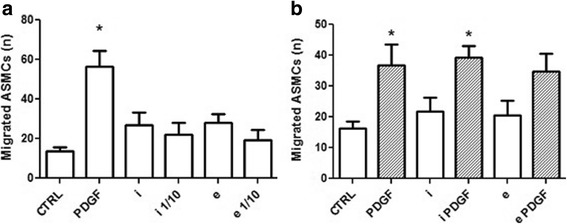
Effect of adipokines on ASMC migration*.* Cell counts are expressed as ‘migrated’ ASMCs at t = 5 h, are plotted as the absolute number (n) of cells counted in the field of view (frame) at 15× magnification for each condition, and data are represented as mean ± SEM. **a** ASMC migration towards serum-free adipocyte media (CTRL), 1 ng/ml PDGF, intrathoracic (*i*) and extrathoracic (*e*) conditioned media, undiluted or diluted (1/10). **b** ASMC migration in 1 ng/mL PDGF-supplemented conditioned media. RM-ANOVA, Bonferroni comparison *P < 0.0001, *n* = 6 (donors)

As previously reported by our group [23, 28], 1 ng/mL PDGF significantly increased ASMC migration (*P* = 0.02; Fig. 2a) and hence used as a positive control to demonstrate chemotaxis of ASMCs in a Transwell® system. Alongside, adipocyte-conditioned media (both depots) did not demonstrate any significant effect on the migrational properties of the ASMCs. Thereafter, the direct effect of the adipocytes on ASMC migration was assessed in a co-culture set-up. Again, 1 ng/mL PDGF stimulated ASMCs showed significant increase in chemotactic response (*P* = 0.0024, Fig. 2b), thereby validating the co-culture system for this study. While 1% FBS-supplemented adipocyte media failed to trigger any chemotaxis in the ASMCs, there was a significant increase observed in presence of 1 ng/mL PDGF, for all conditions (Fig. 2b). There was however, no difference in trend noticed when co-cultured with adipocytes, extra-thoracic or intra-thoracic (Fig. 2b).

### Effects on bovine airway smooth muscle contractility

**Fig. 3:**
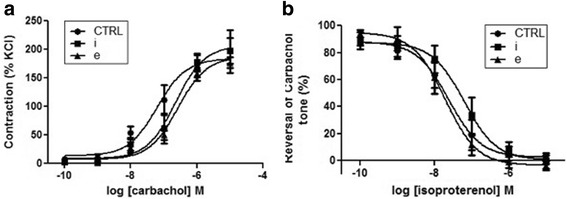
Contractility of bovine tracheal strips in the presence of human adipocyte-conditioned media. Bovine ASM strips were hung in baths filled with Krebs or 1:2 dilutions (in Krebs) of 0% FBS adipocyte media (CTRL), intrathoracic (*i*) & extrathoracic (*e*) adipocyte-conditioned media. The data are expressed as the percent KCl contraction (**a**) or a reversal of the carbachol tone (**b**) with increasing strengths of isoproterenol, mean (%) ± SEM, *n* = 6 (donors)

Adipocyte-conditioned media did not cause any significant changes to the contractile or relaxant responses of bovine tracheal smooth muscle strips (Fig. 3). The half maximal effective concentration (EC_50_) for carbachol, half maximal inhibitory concentration (IC_50_) for isoproterenol and the corresponding maximal responses recorded were statistically insignificant between the pre-treated groups and the control (Table 2). Considering the variability in data, a trend for increase in EC_50_ seen in pre-treated strips cannot be deduced. Again, pre-treatment with intra-thoracic adipocyte-conditioned media showed an increased IC_50_ value. Though insignificant, there was a higher trend noticed for contractions for muscle strips pre-treated with extra-thoracic adipocyte media, with high variability (5.7 ± 2.9% vs. 3.07 ± 1.03% intrathoracic media, *P* = 0.2, Additional file 1: Figure S1, online repository). The data thus suggests the possible differential effects of adipose depots (or sites) on the contractility of airway smooth muscles, but further investigation should be conducted to confirm the same.

**Table 2:** Maximal responses and half maximal effective and inhibitory concentrations

	Carbachol		Isoproterenol	
	EC_50_ (nM)	Max response (%KCl)	IC_50_ (nM)	Max response (%carbachol)
Control	62 ± 38	184 ± 16	24 ± 12	2 ± 5
Intrathoracic	225 ± 110	206 ± 16	62 ± 33	0 ± 6
Extrathoracic	260 ± 62	187 ± 8	19 ± 8	−4 ± 5

EC_50_ and IC_50_ for carbachol and isoproterenol ± pre-treatment with adipocyte-conditioned media. Data presented as mean ± SEM. *n* = 6 (donors)

### Airway smooth muscle cell cytokine synthesis

**Fig. 4:**
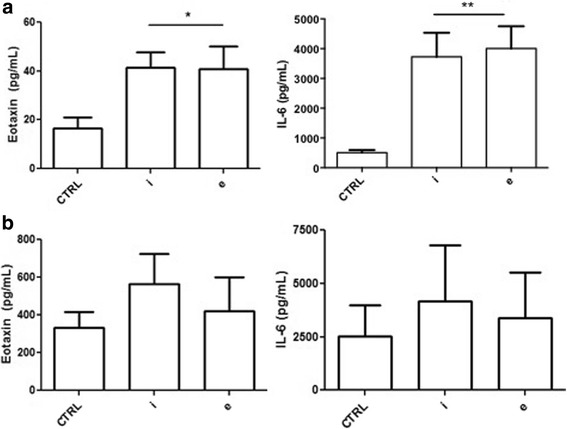
Cytokine synthesis by ASMCs in presence of adipocytes. **a** Eotaxin and IL-6 production by ASMCs when stimulated with serum-free, non-conditioned media (CTRL), intrathoracic (*i*) and extrathoracic (*e*) adipocyte conditioned media for 24 h. **b** represents Eotaxin and IL-6 released by ASMCs when cultured in cell-free 10% FBS supplemented RPMI media (CTRL), intrathoracic (*i*) and extrathoracic (*e*) adipocytes for 3 days. Data represented as mean ± SEM (*n* = 6 donors), repeated measures one-way ANOVA. *indicates significant difference from CTRL, *P* < 0.05. IL-13 and cytomix were used as positive controls for assay validation that allowed significant release of IL-6 (31.98 ± 9.2 ng/ml) and eotaxin (74 ± 19 pg/mL) in by ASMCs respectively (data not plotted)

Eotaxin, IL-6 and TNF-α were measured in ASMC supernatants obtained after three days in co-culture and after 24-h stimulation with adipocyte-conditioned media. To validate the assays, IL-13 (10 ng/mL,) and Cytomix (IL-1β: 5 ng/mL, TNF-α: 30 ng/mL and IFN-γ: 100 ng/mL) were used as positive control for eotaxin, IL-6 and TNF-α release by ASMCs as per previous reports [23]. TNF-α was below detectable limits in the ASMC supernatants for all conditions tested, though a small amount (6 ± 1 pg/mL), could be measured with addition of Cytomix in the control media. After 24-h stimulation with control non-conditioned adipocyte media, the ASMCs secreted 17 ± 11 pg/mL of eotaxin which was significantly increased to 74 ± 19 pg/mL in presence of IL-13 (*P* = 0.0004). Up to 41 ± 16 pg/mL and 41 ± 23 pg/mL of eotaxin was detected when ASMCs were incubated with intra-thoracic and extra-thoracic supernatants respectively, which were statistically significant when compared to the control (*P* = 0.0067, Fig. 4a) but not each other. Again, adipocyte-conditioned media from both depots allowed significant release of IL-6 compared to control (*P* = 0.0031, Fig. 4a). The assay was validated by measuring IL-6 release by ASMCs in presence of cytomix (31.98 ± 9.2 ng/ml, compared to 3.7 ± 0.8 ng/ml, intra-thoracic, and 4.0 ± 0.7 ng/ml, extra-thoracic, *P* = 0.0006, data not plotted). Eotaxin release by ASMCs in co-culture with both intra-thoracic and extra-thoracic adipocytes was greater than that detected with the adipocyte-conditioned media and the serum-free control, but not between the two sites (Fig. 4b). It is evident from the data that adipocytes exhibit the potential to maintain ASMCs at an ‘inflammatory state’, with significant release of two prime pro-inflammatory cytokines, eotaxin and IL-6.

## Discussion

For the first time, we hereby report that human adipocytes (and secreted mediators) isolated from both subcutaneous (extra-thoracic) and visceral (intra-thoracic) adipose sites, do not affect the proliferative or the chemotactic responses of human ASMCs. In addition, they have negligible effect on the contractility, a property crucial to consider the hyper-responsiveness component of asthma. But adipocytes have the capability to stimulate ASMCs to release eotaxin and IL-6, which are pro-inflammatory in nature, thereby contributing to the chronic inflammatory state, archetypal for both asthma and obesity.

While obesity is now recognised as a pro-inflammatory systemic state, airway inflammation is considered to be pivotal component in asthma pathophysiology. However, in a study with 727 adult participants we failed to document any association between BMI and airway inflammation (measured by sputum cell counts) [30]. In a subsequent study, elevated sputum IL-5 and submucosal airway eosinophilia (and not sputum) were documented to be significantly elevated in severe asthmatics with obesity [31]. The detectable increase in IL-5 in the sputum, in the absence of any association with sputum eosinophils, could be attributed to an alternative source/pathway. In the current study, we observed that adipocyte-conditioned media as well as direct culture with adipocytes, stimulated ASM cells towards an inflammatory state (Fig. 4a, b). Adipocyte-conditioned media from both sites accounted for a significant release of both IL-6 and eotaxin from ASMCs post 24 h compared to control unconditioned media. Comparative to the conditioned media, a significant increase in eotaxin release was seen in the co-cultures for all tested conditions (*P* = 0.0003, Two-way ANOVA), whereas IL-6 release was highly variable. The increase of eotaxin release in the co-culture could be attributed to the presence of 10% FBS in the media, which was absolutely required for sustenance of the co-culture. Nevertheless, the result provides initial evidence that adipocytes directly affect and maintain the inflammatory state of the ASMs. Increased eotaxin release from the adipocytes, classically known to recruit eosinophils into the tissue, could explain the increased sputum eosinophils into the submucosa and the associated IL-5 seen by Desai., et al. [31]. In addition, a recent study demonstrated that the newly described tissue resident group 2 innate lymphoid cells (ILC2s) were activated upon calorie-intake, with secretion of IL-5 in situ [32]. In particular, ILC2s resident in the visceral adipose tissue (VAT) sustained eosinophilia [33]. Further, we recently documented increased abundance of IL5^+^ ILC2s in the sputum of severe asthmatics compared to mild and healthy. Therefore, the role of these cells in substantiating the inflammatory state initiated by the adipocytes, and further contributing to asthma severity requires investigation.

The relationship between the documented increase in AHR with a higher adiposity index can be adequately explained by the changes in breathing mechanics, rather than ‘inflammation’ [6, 34]. Shallow breathing (increased frequency, lower tidal volumes) and reduced deep inspirations in obese individuals lead to reduced lung volumes which has a profound effect on the ASM unloading, and thereby the AHR to methacholine [35]. This can explain the innate AHR observed in non-asthmatic obese population [15] but not the animal models [7, 17], since the mice are ventilated at fixed tidal volume and ASM unloading does not occur during AHR measurements. If it is to be assumed that the mouse models closely mimic the disease progression in humans, then it is highly likely that changes in the ASM have a different etiology independent of the altered breathing mechanics. Consequently, the likelihood of increased adipose depots and their secreted products modulating the ASM biology was the main scope of this study.

Countering this study question, the data fails to show any significant modulatory effects of adipocytes and its secreted products on the ASM proliferation (Fig. 1), migration (Fig. 2) or contractility of bovine muscle strips (Fig. 3). These observations could therefore explain the results of Sutherland et al., where no apparent association between leptin and adiponectin levels with asthma was documented [10]. Furthermore, the first randomised trial of a weight-loss regimen on improvement of asthma symptoms, showed a weak association of serum leptin with the measured total lung capacity and hyperreactivity pre- and post-intervention [36].

Only low levels of leptin could be detected in the adipocyte-conditioned supernatants (data not shown) by ELISA, when compared to previously reported studies [37, 38]. This is possibly due to differences in culture methods or likely due to lower concentrations of isolated adipocytes. Previously, eotaxin production by ASM stimulated by 100 ng/mL leptin (48 ± 46 pg/mL, *n* = 5) was reported to be lesser that that produced by unstimulated cells (67 ± 74 pg/mL, *n* = 5) [23]. This is contrary to the significant amount of eotaxin production by ASM cultured with adipocyte-conditioned media containing a cocktail of adipokines (41 ± 16 pg/ml vs. control 17 ± 11 pg/ml, *P* = 0.0067, *n* = 6). It is therefore evident, that any plausible inflammatory mechanism that might affect the ASM in obese is factored in by one or more of the mediator(s) released and not just by leptin alone. In fact, it is increasingly being recognised that the stromal vascular fraction and the tissue matrix constituting the ‘non-fat cells’ of the adipose tissue contribute significantly to the inflammatory profile [6, 8, 39]. There is an increase in macrophages present in the adipose tissue of the obese asthmatic individuals that have been demonstrated to release the major proportion of TNF-α and IL-6, in comparison to the adipocytes [6, 8]. This is further confirmed since TNF-α was undetectable in our co-cultures with ‘isolated’ adipocytes. This warrants a need to further investigate the effect of the ‘non-fat’ cells, comprising the macrophages and tissue matrix components, in addition to the adipocytes, in modulating ASM towards a diseased phenotype. A study design with supernatants conditioned by the adipose tissue explants instead of isolated adipocytes might be more appropriate to investigate the defined objectives in the future. In addition, we acknowledge that measuring only leptin was a drawback of the current study, when an adipokine array is commercially available (adipokine microarrays should therefore be used in future experiments).

Considering the potential difference in mediators secreted at different sites [26, 40, 41], the study for the first time addressed how intrathoracic and extrathoracic adipocytes could affect ASM biology. Intrathoracic adipose tissue was chosen as the visceral depot nearest the lung and extrathoracic samples were extracted from subcutaneous sites in the chest wall. In particular to the released adipokines, the intrathoracic samples showed a trend towards increased synthesis of eotaxin and IL-6 levels. Similar observations by Fain, et al.*,* were reported where the adipokine synthesis by visceral fat was higher in comparison to subcutaneous fat, even though the former was mostly abdominal in origin [38]. In our current study however, for all the experimental outcomes, there was no significant difference encountered in between the two depots which is in accordance with the recent observations by Fenger and co-workers, that higher risk of asthma with increasing obesity is independent of the distribution of adiposity [42]. Again, a recent study reported similar trends for leptin and adiponectin expression by visceral and subcutaneous adipose tissue [12]. In the light of the current discussion, it is important to indicate that the current study design did not distinguish between the BMI of the donors (out of the six donors, four had BMI > 32, one was overweight with BMI of 28.6 and another normal at 23). The authors remain aware that there are major differences in the adipocytes of lean and obese individuals with respect to morphology and adipokine synthesis. Again, sex of the donor was not considered in the study design either, even though sexual dimorphisms in the underlying mechanisms of obesity-asthma have been observed [6, 40, 43]. The initial lay-out of the study was to assess the immediate effect of adipocytes from visceral and subcutaneous sources (from the vicinity of the airways) on ASM, without any further co-variables. Till date there are few mechanistic studies that investigate the direct influence of adipose tissue on the human ASM. Therefore, the data reported here is unique and provides direction to future studies.

## Conclusion

In summary, the current study provides the first evidence of the modulatory effects of human adipocytes on human airway smooth muscle biology. We demonstrate that adipocytes per se do not have a plausible role towards modulating the ASM towards hyperplasia, hypertrophy or hyperreactivity, but exhibits a potential to induce and maintain a low-grade inflammatory profile. It remains to be seen if this is likely to contribute to the tissue eosinophilic inflammation reported in obese patients with asthma.

## Additional file

Additional file 1: **Figure S1.** Contractility of bovine tracheal strips in the presence of human adipocyte conditioned media. Contractions are expressed as the percent KCl contraction. Bovine ASM strips were hung in baths filled with Krebs or 1:2 dilutions (in Krebs) of 0% FBS adipocyte media (CTRL), intrathoracic (i) & extrathoracic (e) adipocyte-conditioned media. Data presented as means ± SEM. *P* > 0.05, *n* = 6. (DOCX 22 kb)

## Abbreviations

AHR: Airway hyper-responsiveness
ASM: Airway smooth muscle
BMI: Body mass index
EC_50_: Half maximal effective concentration
FBS: Fetal bovine serum
IC_50_: half maximal inhibitory concentration
IL: Interleukin
PDGF: Platelet-derived growth factor
TNF*-α*: Tumour necrosis factor alpha
