# Supplementary material for: Modulation of human airway smooth muscle biology by human adipocytes
Source: Respir Res. 2018 Feb 27;19:33. doi: 10.1186/s12931-018-0741-z (PMC5830317; doi:10.1186/s12931-018-0741-z)
Supplement: Supplementary file 1 — Figure S1. Contractility of bovine tracheal strips in the presence of human adipocyte conditioned media. Contractions are expressed as the percent KCl contraction. Bovine ASM strips were hung in baths filled with Krebs or 1:2 dilutions (in Krebs) of 0% FBS adipocyte media (CTRL), intrathoracic (i) & extrathoracic (e) adipocyte-conditioned media. Data presented as means ± SEM. P > 0.05, n = 6. (DOCX 22 kb) [file 12931_2018_741_MOESM1_ESM.docx]

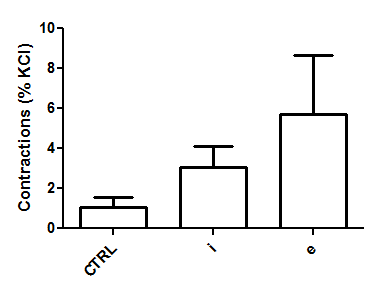


**Figure S1:** Contractility of bovine tracheal strips in the presence of human adipocyte conditioned media. Contractions are expressed as the percent KCl contraction. Bovine ASM strips were hung in baths filled with Krebs or 1:2 dilutions (in Krebs) of 0% FBS adipocyte media (CTRL), intrathoracic (i) & extrathoracic (e) adipocyte-conditioned media. Data presented as means ± SEM. P > 0.05, n=6
